# Artificial intelligence in infection prevention and control education: toward intelligent, real-time clinical learning systems

**DOI:** 10.3389/fpubh.2026.1927532

**Published:** 2026-08-25

**Authors:** Yu-long Cao, Na Liu, Fang Wang, Jiao Shan, Xiao-yuan Bao, Meng Jin, Wei Huai, Yi-cheng Jin, Yi-xi Jin, Zhi-ning Zhang, Ji-qiu Kuang

**Affiliations:** 1 Department of Hospital-Acquired Infection Control, Peking University People's Hospital, Beijing, China; 2 Department of Quality Management, Weihai Municipal Hospital, Cheeloo College of Medicine, Shandong University, Weihai, Shandong, China; 3 Department of Hospital Infection Management, The Second Affiliated Hospital of Inner Mongolia Medical University, Hohhot, Inner Mongolia, China; 4 Department of Hospital-Acquired Infection Control, Beijing Jishuitan Hospital, Capital Medical University, Beijing, China; 5 Medical Informatics Center, Institute of Advanced Clinical Medicine, Peking University, Beijing, China; 6 Department of Emergency, Peking University Third Hospital, Beijing, China; 7 School of General Studies, Columbia University, New York, NY, United States; 8 Khoury College of Computer Science, Northeastern University, Seattle, WA, United States; 9 Graduate School of Medicine, Kyoto University, Kyoto, Japan

**Keywords:** artificial intelligence, clinical learning, education, infection control (IC), infection prevention

## Abstract

Artificial intelligence (AI) is increasingly being explored in infection prevention and control (IPC) education as a means of addressing limitations of conventional training, including limited scalability, delayed feedback, and subjective competency assessment. This mini review summarizes emerging applications of AI-enabled technologies in IPC education and examines their potential educational benefits, limitations, and implementation considerations. Current evidence includes applications of computer vision for behavioral assessment, AI-integrated virtual and extended reality for immersive and adaptive learning, and large language models for knowledge support and interactive learning. Available studies suggest that AI-assisted interventions may improve procedural performance, learner engagement, feedback efficiency, and selected measures of knowledge or self-efficacy. However, the evidence remains heterogeneous, with substantial variation in AI modalities, learner populations, study designs, outcome measures, and follow-up periods. Many studies are based on small samples or single-center settings and primarily evaluate short-term educational outcomes, limiting conclusions regarding sustained competency acquisition or translation into clinical practice. Evidence linking AI-assisted IPC education to reductions in healthcare-associated infections remains particularly limited. Important challenges also include workforce readiness, infrastructure and interoperability, privacy and governance, and potential cognitive burden associated with immersive technologies. Overall, AI shows promise as an enabling component of adaptive, data-supported, and feedback-oriented IPC education, but its educational and clinical value requires further validation through larger, multicenter, longitudinal, and implementation-focused studies.

## 1 Introduction

The integration of artificial intelligence (AI) into infection prevention and control (IPC) education has emerged as a response to persistent structural deficiencies in conventional training systems. Across the existing literature, three interrelated constraints are consistently emphasized: limited training capacity and scalability, inadequate mechanisms for real-time and objective performance feedback, and the absence of standardized, scalable frameworks for competency assessment across heterogeneous healthcare environments (1–3).

Despite the widespread reliance on traditional didactic approaches, evidence indicates that such models remain inherently constrained by limited adaptability and delayed feedback loops, both of which hinder iterative skill acquisition and the refinement of procedural competencies. In contrast, recent technological advances demonstrate the feasibility of AI-driven systems capable of real-time procedural evaluation, particularly through the transformation of complex clinical behaviors into quantifiable and continuously monitored performance metrics. At the system level, emerging synthesis studies further highlight the development of structured implementation frameworks that assess institutional readiness, operational risks, and governance requirements associated with AI adoption in IPC settings. These developments suggest that AI should not be conceptualized as a supplementary enhancement to existing educational paradigms. Rather, it represents a structural transition toward data-driven, adaptive, and continuously optimized competency development systems in infection prevention education.

To provide an analytical perspective beyond categorizing AI applications by technology type, we propose a conceptual framework describing how AI fundamentally transforms infection prevention and control (IPC) education. Rather than functioning as isolated educational tools, AI technologies reshape IPC learning through three interconnected mechanisms: intelligent knowledge augmentation, real-time competency assessment, and adaptive simulation-based learning. First, natural language processing and large language models extend traditional knowledge delivery by enabling dynamic information retrieval, context-aware explanation, and personalized learning support, thereby transforming education from static content transmission toward interactive knowledge augmentation. Second, computer vision and sensor-based technologies enable continuous and objective assessment of procedural behaviors, overcoming the limitations of conventional instructor-dependent evaluation and delayed feedback. Third, virtual reality, extended reality, and emerging AI-driven adaptive learning environments facilitate immersive scenario-based training that can be tailored to individual learner characteristics and clinical contexts. Collectively, these mechanisms represent a transition from instructor-centered, standardized training models toward intelligent learning ecosystems characterized by continuous feedback, personalization, and competency-based progression. This framework provides the conceptual foundation for interpreting the subsequent evidence on AI applications in IPC education.

In this review, artificial intelligence (AI) refers to computational systems capable of performing functions that typically require human-like cognitive processes, including learning from data, pattern recognition, prediction, language processing, perception, or adaptive decision-making. Importantly, we distinguish AI-enabled educational interventions from conventional digital or simulation technologies. Virtual reality (VR), extended reality (XR), and computer-based simulation are primarily educational delivery and experiential-learning modalities and do not constitute AI by themselves. We therefore use the term “AI-enabled VR/XR or simulation” only when these environments incorporate AI-based functions, such as computer vision for automated performance assessment, machine learning for learner-state or behavior prediction, natural language processing or large language models for interactive feedback, or adaptive algorithms that dynamically modify learning content according to learner performance. This distinction is important because the educational effects of conventional immersive simulation cannot be attributed to AI unless an AI component is explicitly incorporated and contributes to the intervention.

## 2 Literature search strategy

A focused literature search was conducted to identify recent evidence regarding artificial intelligence-enabled technologies in infection prevention and control (IPC) education. PubMed/MEDLINE was searched from inception to March 31, 2026. using combinations of the following terms: (“artificial intelligence” OR “machine learning” OR “deep learning” OR “large language model” OR “natural language processing” OR “computer vision”) AND (“infection prevention” OR “infection control” OR “hand hygiene” OR “personal protective equipment” OR “healthcare education” OR “medical education”). Additional relevant studies were identified through reference screening of included articles and recent reviews.

Studies published in English that evaluated AI-enabled educational interventions, AI-supported assessment systems, or AI-based learning assistance in healthcare education or IPC-related training were considered eligible. Studies focusing solely on non-AI immersive technologies, including virtual reality, extended reality, or simulation-based education without identifiable AI functions, were excluded from the AI evidence table but were considered as contextual evidence where appropriate. For the evidence summary table, we selected representative primary studies that provided sufficient information regarding the AI technology, educational application, study population, and learning-related outcomes. Reviews, commentaries, and studies without a clearly described AI component were not included as primary evidence.

## 3 AI-enabled transformation of IPC education: linking educational functions with enabling technologies

The educational impact of AI in IPC training is determined not solely by individual technologies but by how specific technological capabilities address distinct educational challenges. Therefore, AI applications in IPC education should be interpreted through the interaction between educational functions and enabling technologies. For example, computer vision transforms procedural training by converting observable behaviors into objective performance indicators, whereas extended reality creates immersive environments for experiential skill acquisition, and large language models provide cognitive augmentation through contextualized knowledge support. Accordingly, the following sections integrate technological mechanisms with their corresponding educational applications to illustrate how AI reshapes IPC competency development.

These educational approaches should be distinguished according to the role of AI within the learning environment. Extended reality systems primarily provide immersive environments for experiential learning, whereas AI-enabled XR systems incorporate additional computational functions, such as automated performance assessment, physiological-state recognition, conversational interaction, or adaptive content delivery. Similarly, computer vision algorithms can transform an otherwise conventional simulation environment into an AI-enabled training system by analyzing procedural sequences and identifying deviations from recommended practices. Thus, in this review, immersion and simulation are considered educational modalities, whereas AI refers specifically to the computational capabilities that enable automated perception, assessment, prediction, interaction, or adaptation. By integrating simulation and automated assessment, AI transforms PPE education from episodic instructor observation into a continuous competency development process. On one hand, virtual reality systems enable high-fidelity replication of clinical scenarios, allowing learners to repeatedly practice donning and doffing procedures in a risk-free setting, thereby strengthening procedural memory through structured repetition and experiential reinforcement (4). On the other hand, computer vision–based systems deployed in real-world clinical environments extend training into operational contexts by detecting unsafe PPE behaviors and delivering immediate corrective feedback, effectively shifting the pedagogical model from retrospective assessment to *in situ* performance correction (5). The convergence of these approaches reflects an emerging hybrid training architecture in which simulated cognitive rehearsal and real-time clinical reinforcement operate within a continuous feedback loop, enhancing both skill acquisition and behavioral consolidation.

Extended reality (XR) technologies are primarily immersive educational modalities rather than AI technologies *per se*. They provide computationally mediated environments characterized by spatial mapping, interactive feedback mechanisms, and layered simulation architectures. When combined with AI components, however, XR environments can support functions that extend beyond immersion, including automated assessment, learner-state recognition, conversational interaction, and adaptive learning. Contemporary evidence highlights the central role of head-mounted display systems in constructing immersive digital overlays that merge physical and virtual clinical spaces, thereby enabling experiential learning within controlled yet highly realistic risk environments (6). Within this spatial computing framework, IPC training is increasingly reconceptualized as an embodied learning process in which procedural knowledge is acquired through interaction within simulated clinical ecologies rather than passive instruction, thereby enhancing both cognitive engagement and procedural retention. Recent advances have further expanded XR-based education toward integrated AI-enhanced mixed reality (MR) learning systems. Sepanloo et al. developed and pilot-tested an AI-enhanced MR training platform for nursing education that integrated immersive simulation with conversational artificial intelligence to facilitate interactive learning and adaptive learner support (7). This emerging paradigm illustrates how AI can transform XR environments from static simulation spaces into responsive educational ecosystems capable of providing personalized feedback and learner-centered interaction. Although current studies remain at an early validation stage, AI-MR platforms represent a promising direction for future IPC education by combining immersive procedural practice with intelligent assessment and feedback mechanisms.

A comparable methodological transition is evident in hand hygiene education, where traditional compliance-based monitoring is being progressively replaced by fine-grained behavioral analytics. Machine learning frameworks capable of identifying and validating discrete handwashing steps in real time enable a more granular assessment of procedural fidelity beyond binary compliance indicators (8). In parallel, convolutional neural network–based approaches have demonstrated performance comparable to expert human evaluators in assessing hand hygiene behaviors in clinical environments, thereby reinforcing the feasibility of automated, micro-level behavioral auditing (9). This evolution signals a conceptual shift in hand hygiene education from frequency-centered surveillance models toward precision-oriented behavioral reconstruction and continuous performance profiling.

Computer vision represents the perceptual layer of this ecosystem and has undergone a clear evolution from early experimental prototypes to sophisticated multimodal architectures. Initial studies demonstrated the feasibility of video-based automated monitoring of hand hygiene behaviors in clinical wards, providing an early proof-of-concept for machine-assisted behavioral tracking in IPC contexts (10). Building on this foundation, more recent advances have integrated multimodal sensing frameworks capable of combining visual, spatial, and contextual data streams to enable continuous monitoring and structured performance reporting, particularly in high-acuity environments such as intensive care units (11). This progression reflects a shift from isolated gesture recognition systems toward context-aware environmental intelligence capable of interpreting complex clinical workflows as integrated behavioral sequences rather than discrete actions.

Beyond frontline clinical practices, AI applications in environmental cleaning and disinfection training have further expanded the scope of IPC education to include ancillary healthcare personnel. Evidence from intervention-based studies suggests that AI-assisted feedback systems incorporating multimodal visual and auditory guidance may improve environmental cleaning performance and task execution accuracy. For example, AI-supported training approaches have demonstrated measurable improvements in procedural performance compared with conventional training methods, although reported outcomes vary depending on the target population, assessment criteria, and implementation context (12). Therefore, current evidence supports the feasibility of AI-enhanced feedback for IPC skill development, while larger-scale validation studies remain needed. This expansion reflects a broader systemic reconfiguration in which IPC training is no longer confined to clinical decision-makers but is increasingly distributed across the entire healthcare delivery workforce, reinforcing a more inclusive and system-wide infection control paradigm.

Extending beyond procedural domains, large language models (LLMs) introduce a fundamentally different layer of AI application focused on cognitive augmentation and decision support. Accordingly, the educational role of LLMs should be conceptualized as cognitive augmentation rather than autonomous expertise replacement. In IPC education, LLMs may provide value by supporting knowledge retrieval, generating practice scenarios, facilitating learner questioning, and promoting reflective reasoning. However, their limitations in complex, high-stakes scenarios indicate that LLM-based educational systems should incorporate expert supervision, retrieval-augmented knowledge sources, and structured validation mechanisms. Rather than providing definitive clinical recommendations, LLMs may be most appropriately positioned as interactive learning partners that enhance reasoning processes while maintaining human oversight for critical decision-making (13). Further cognitive evaluation using structured reasoning frameworks has revealed systematic limitations in LLM performance when addressing high-risk IPC scenarios such as needlestick injury management and PPE removal decision pathways (14). These findings position LLMs as promising yet intrinsically imperfect cognitive assistants whose integration into IPC education and clinical workflows must be carefully governed, with explicit requirements for expert oversight and contextual validation. Complementing perceptual and experiential layers, natural language processing (NLP) and large language models (LLMs) constitute the semantic intelligence layer of AI-driven IPC education. Recent advances demonstrate that generative AI systems are capable of extracting infection-related variables from large-scale unstructured clinical narratives, including electronic health records and clinical documentation, thereby enabling both knowledge synthesis and predictive modeling in public health applications (15). This functionality effectively positions LLMs as dynamic semantic intermediaries that translate static clinical text into structured, context-aware knowledge representations, supporting adaptive learning systems and expanding the informational backbone of IPC education.

## 4 Effectiveness, evidence quality, and educational implications of AI-assisted IPC training

Within the proposed framework, the educational value of AI should be evaluated according to its ability to support feedback, learner adaptation, and competency development rather than assumed from the presence of an AI component alone. Available studies suggest that AI-assisted IPC training may improve selected aspects of technical performance, behavioral compliance, learner engagement, and self-efficacy. However, these findings should be interpreted cautiously because the evidence base remains heterogeneous with respect to AI modality, learner population, intervention design, comparator, outcome definition, and follow-up duration.

Table 1 summarizes representative evidence across several levels of AI-enabled and immersive IPC education, ranging from clinical AI-assisted training interventions and automated behavioral assessment to LLM knowledge evaluation, technical validation studies, and conventional VR-based education. These studies are therefore not directly comparable in terms of intervention intensity, population, outcome definition, or evidentiary level. Importantly, conventional VR studies are presented separately from AI-enabled interventions because immersion alone does not constitute an AI function. The table also distinguishes participant-level samples from technical evaluation units, such as hand-hygiene events, model–question responses, and procedural steps, to avoid interpreting technical validation datasets as educational cohorts.

**Table 1:** Representative studies evaluating AI-assisted, AI-integrated, and immersive technologies in infection prevention and control education.

Study	Technology platform	Specific AI function	Educational application	Study design and participants	Main outcomes
Segal et al., 2023 (16)	AI-assisted PPE assessment system	Computer vision-based recognition of PPE donning and doffing behaviors with automated performance feedback	PPE competency training and procedural error correction	Prospective educational evaluation among healthcare trainees using an AI-supported PPE assessment platform	AI-assisted assessment enabled automated identification of procedural deviations and supported targeted feedback during PPE training
Preda et al., 2025 (5)	AI-enhanced PPE training platform (SXR AI-PPE)	Computer vision-based PPE event recognition and automated assessment of donning/doffing procedures	PPE donning and doffing education and remediation training	Prospective intervention study including 293 participants from healthcare-related disciplines	AI-guided assessment identified PPE-related errors and supported improvement after remediation; unguided doffing failures decreased after training exposure
Quiñones-Romero et al., 2025 (8)	Machine learning-based hand hygiene assessment system	Machine learning-based action recognition and automated evaluation of hand hygiene performance	Hand hygiene competency assessment and behavioral feedback	AI-based evaluation study using recorded hand hygiene performance data	Machine learning models demonstrated the feasibility of automated recognition and standardized assessment of hand hygiene behaviors
Simioli et al., 2024 (11)	AI-supported IPC educational/monitoring platform	AI-assisted monitoring and reporting support (specific algorithmic approach requires further clarification)	IPC practice monitoring and educational feedback	Healthcare professional training/implementation evaluation	AI-supported approaches improved standardization of IPC monitoring processes, although further validation is required
Jawanpuria et al., 2024 (14)	Large language model (ChatGPT)	Generative AI-based natural language generation and IPC knowledge question answering	IPC knowledge assessment and learner support	Evaluation of 110 IPC-related questions assessed by infection control experts	ChatGPT achieved an overall expert rating score of 4.33; 63.6% of responses were rated above acceptable quality, while expert verification remained necessary
Abosi et al., 2025 (13)	Large language model-based educational platform	Natural language generation and guideline-based question answering	IPC-related information retrieval and educational support	Comparative evaluation of multiple LLMs for IPC-related queries	LLMs provided useful IPC information support but demonstrated limitations in accuracy, consistency, and complex clinical reasoning scenarios

At the procedural level, several studies have reported improvements in technical accuracy and behavioral performance following AI-enabled feedback interventions, particularly during high-risk procedures such as PPE donning and doffing (16). However, the available studies differ in intervention design, participant characteristics, assessment methods, and comparison conditions, making direct comparison difficult. Unlike traditional summative assessment, AI-enabled systems can facilitate formative feedback by identifying performance deviations during or immediately after task execution. This closed-loop approach may help learners recognize errors, modify behaviors, and reinforce correct practices during training. Nevertheless, whether these short-term improvements translate into durable competency retention or improved adherence during routine clinical practice remains uncertain.

Beyond technical performance, immersive learning approaches may influence higher-order cognitive and psychological outcomes. Virtual reality–based IPC education has been associated with improvements in knowledge acquisition, self-efficacy, and perceived clinical preparedness in some studies (17). However, these findings primarily demonstrate the educational potential of immersive simulation and should not be interpreted as evidence of an independent effect of AI unless an AI component was explicitly incorporated and evaluated. Where AI is integrated into VR or XR environments, its potential contribution may arise from adaptive feedback, automated assessment, physiological-state recognition, or personalized scenario delivery. Further comparative studies are needed to disentangle the effects of immersion, simulation, and AI-enabled adaptation.

The potential benefits of immersive and AI-assisted training should nevertheless be interpreted in the context of the available comparative evidence. Omori et al. evaluated a virtual reality-based infection control training program among medical students (21 students in each group) and reported higher post-training OSCE scores in the VR group than in the lecture group (median total score: 12 [IQR 9.5–12] vs. 9 [IQR 8–12], *P* = 0.024) (18). This finding supports the potential value of immersive simulation for procedural learning, but it does not by itself establish an independent effect of AI. Similarly, systematic reviews of VR-based medical education have reported favorable effects on procedural skills and learner engagement while emphasizing substantial heterogeneity in study designs and outcome measures (19). Taken together, these findings indicate that immersive and AI-assisted approaches may provide useful mechanisms for competency-oriented IPC education, but the relative contribution of AI remains insufficiently established and warrants direct comparative evaluation.

## 5 Challenges, implementation considerations, and future research priorities

These limitations also explain why the effectiveness findings reported in educational studies should be interpreted cautiously. The current evidence suggests promising improvements in learning outcomes; however, the field has not yet established whether AI-enhanced training consistently leads to long-term competency retention, sustained behavioral improvement, or measurable reductions in healthcare-associated infections.

Despite the growing body of evidence supporting AI-assisted IPC education, its large-scale implementation remains constrained by a combination of institutional, methodological, and human-centered challenges. These limitations not only hinder the widespread adoption of AI technologies but also restrict their translation from experimental educational tools into routine clinical practice.

At the institutional level, successful implementation depends on organizational readiness rather than technological availability alone. Current evidence indicates that insufficient financial investment, inadequate AI literacy among healthcare professionals, and persistent interoperability challenges between AI platforms and existing hospital information systems continue to impede large-scale deployment (20). More importantly, the limited preparedness of healthcare organizations to integrate AI into established educational and clinical workflows highlights a critical disconnect between technological innovation and implementation capacity. These findings suggest that sustainable adoption will require coordinated investments in digital infrastructure, workforce development, and institutional governance alongside continued technological advancement.

Beyond implementation barriers, the current evidence base remains insufficient to fully establish the long-term educational and clinical value of AI-assisted IPC training. Most published studies are characterized by relatively small sample sizes, single-center designs, and short follow-up periods, with outcome measures primarily focused on immediate improvements in procedural performance or behavioral compliance rather than patient-centered clinical endpoints, such as reductions in healthcare-associated infections. Consequently, an important translational gap persists between educational effectiveness and measurable improvements in infection prevention outcomes, limiting the generalizability and external validity of the existing literature (21).

The implementation of computer-vision-based real-time behavioral tracking systems also raises important ethical and governance considerations that differ according to whether the technology is used in a controlled training environment or deployed in routine clinical practice. In training settings, monitoring should be explicitly framed as an educational activity, with learners informed about what data are collected, whether individuals can be identified, how performance data are interpreted, who can access the data, and how long the data will be retained. Where possible, training systems should minimize identifiability by collecting only data necessary for competency assessment and using de-identification or pseudonymization when individual identification is not required. In contrast, clinical deployment may involve continuous monitoring of healthcare workers and potentially patients or other individuals, creating substantially greater risks related to privacy, surveillance, and secondary use.

The distinction between primary and secondary use of surveillance data is particularly important. Data initially collected to provide formative educational feedback should not automatically be repurposed for staff performance ranking, disciplinary action, employment decisions, regulatory reporting, commercial purposes, or model development without a clearly defined legal and governance basis and appropriate transparency. Retention periods should also be proportionate to the educational purpose, with predefined policies specifying data ownership or stewardship, access permissions, retention duration, deletion procedures, and conditions for reuse. These safeguards are particularly relevant for video, physiological, and interaction data because seemingly non-identifying information may become identifiable when linked with timestamps, locations, staff rosters, or other institutional records.

Automated assessments should also be contestable. Learners and educators should have a clear mechanism to review an AI-generated assessment, access an appropriate explanation or supporting evidence, request human review, and correct demonstrably erroneous records. This is especially important when automated assessments may affect certification, remediation, professional evaluation, or employment-related decisions. AI-generated scores should therefore be treated as reviewable evidence rather than unquestionable determinations. A human-in-the-loop governance model should define who is responsible for resolving disputed assessments, how disagreements are documented, and how corrected assessments are incorporated into the learner's record. Such mechanisms can preserve the educational value of automated monitoring while reducing the risk that surveillance technologies become instruments of opaque or punitive performance management.

Equally important are the challenges arising from human–AI interaction. Although immersive technologies and intelligent feedback systems have demonstrated considerable educational potential, their effectiveness ultimately depends on user acceptance and cognitive usability. Device complexity, unfamiliar interaction modalities, and increased cognitive demands may inadvertently distract learners from core educational objectives, particularly among novice users with limited digital experience. Existing evidence indicates that such usability barriers can reduce learning efficiency and hinder technology adoption, emphasizing that future AI-enabled educational systems should prioritize human-centered design, intuitive interfaces, and seamless integration into routine clinical training environments (22). These challenges suggest that the future development of AI-assisted IPC education should be guided not only by technological innovation but also by rigorous clinical validation, implementation science, and user-centered design. Addressing these complementary dimensions will be essential for translating promising technological advances into sustainable educational practice and meaningful improvements in infection prevention.

Recent studies have begun to provide objective physiological evidence regarding learner responses during immersive training. Sepanloo et al. investigated physiological stress responses among nursing students participating in MR-based simulation training using biosensor-derived indicators, including heart rate, electrodermal activity, and skin temperature (23). By applying machine learning approaches to physiological signals, the study demonstrated the feasibility of real-time stress-state classification during immersive learning. These findings suggest that AI-enhanced MR systems may not only deliver immersive educational experiences but also enable monitoring of learner cognitive and physiological states. However, physiological stress responses should not be interpreted solely as indicators of excessive cognitive burden, as moderate stress may also reflect engagement and realistic simulation experiences. Future research should clarify how adaptive AI feedback can optimize the balance between learning challenge and cognitive workload.

Future research should move beyond evaluating educational outcomes alone and establish whether AI-enhanced IPC training can generate measurable improvements in clinical practice. A key priority is the development of implementation studies that connect competency acquisition with infection prevention outcomes. For example, multicenter cluster-randomized trials comparing AI-enhanced IPC training with conventional education could evaluate not only learner performance but also downstream indicators, including hand hygiene compliance, healthcare-associated infection rates, outbreak response efficiency, and long-term retention of IPC competencies. Such studies should incorporate longitudinal follow-up and implementation science frameworks to determine how educational improvements translate into real-world infection prevention impact.

The implementation of AI-driven IPC education will also require context-specific strategies across different healthcare environments. High-resource healthcare systems may benefit from advanced AI capabilities integrated with electronic health records, simulation platforms, and automated surveillance systems; however, challenges related to interoperability, governance, and workforce acceptance remain. In contrast, low-resource settings may face limitations in digital infrastructure, financial investment, and technical expertise. Nevertheless, lightweight AI solutions, mobile-based learning platforms, and locally adapted models may provide opportunities to expand IPC education capacity where traditional training resources are limited. Therefore, global adoption should emphasize adaptable and equitable AI ecosystems rather than a one-size-fits-all technological approach.

The rapid emergence of generative AI introduces additional requirements for educational governance and ethical oversight. Before integration into IPC training systems, LLM-based tools should undergo systematic evaluation for accuracy, bias, transparency, and safety. Appropriate safeguards should include expert-supervised deployment, retrieval-augmented generation using validated IPC knowledge sources, continuous performance monitoring, and clear delineation between educational assistance and clinical decision-making. Establishing governance frameworks will be essential to ensure that generative AI enhances learning while maintaining professional accountability and patient safety.

The Chinese healthcare context provides an important example for understanding both the opportunities and challenges of AI-enabled IPC education. China has experienced rapid expansion of digital health infrastructure, large-scale hospital networks, and increasing adoption of intelligent healthcare technologies, creating opportunities for scalable AI-supported training models. However, differences in hospital capacity, regional healthcare resources, and workforce characteristics highlight the importance of adaptable implementation strategies. Lessons from China suggest that successful adoption requires not only technological innovation but also integration with national IPC priorities, professional training systems, and local healthcare workflows. These experiences may provide valuable insights for other countries seeking to develop sustainable and context-sensitive AI-enabled IPC education strategies.

## 6 Conclusions and future perspectives

The available evidence suggests that artificial intelligence is emerging as a potentially important enabler of IPC education by supporting data-informed feedback, automated assessment, interactive knowledge support, and adaptive learning (24). Across the currently available studies, AI-assisted and AI-integrated technologies have shown potential to improve selected educational outcomes, including procedural performance, learner engagement, feedback efficiency, and self-efficacy. However, the evidence remains heterogeneous and should not be interpreted as demonstrating a uniform or independent effect of AI across IPC educational settings. In particular, evidence from conventional VR and simulation should be distinguished from evidence generated by AI-enabled interventions, and the contribution of AI components is not always separately evaluated.

As AI technologies continue to mature, IPC education may evolve toward more personalized, context-aware, and competency-oriented learning environments. Emerging applications, including adaptive virtual simulation and AI-supported interactive learning, provide promising opportunities to tailor training to different clinical specialties, healthcare settings, and learner characteristics. Nevertheless, these applications remain at varying stages of development and validation (25). Their educational value will depend not only on technological capability but also on the quality of instructional design, the appropriateness of outcome measures, and the degree of integration with established IPC training systems.

Future research should therefore move beyond proof-of-concept demonstrations and short-term educational outcomes toward rigorous evaluation of effectiveness, implementation, and clinical relevance. Multicenter and longitudinal studies, preferably using well-defined comparators and standardized competency outcomes, are needed to determine whether AI-assisted training produces durable improvements in knowledge, procedural competence, and clinical behavior. Where AI is integrated with VR, XR, or simulation, study designs should also seek to isolate the contribution of the AI component from the effects of immersion, repetition, and simulation itself (26, 27). Ultimately, evidence linking AI-assisted IPC education with downstream outcomes such as sustained adherence, outbreak response performance, or reductions in healthcare-associated infections will be necessary to establish its broader clinical and public health value.

AI should therefore be regarded not as a proven replacement for conventional IPC education, but as an emerging enabling technology that may augment human-led training when appropriately designed, validated, and governed. By combining expert supervision with adaptive feedback, simulation, and data-supported learning, AI has the potential to strengthen IPC workforce development while maintaining educational quality, professional accountability, privacy, and patient safety.
